# Influence of Honey bee Nutritive Jelly Type and Dilution on its Bactericidal Effect on *Melissococcus plutonius*, the Etiological Agent of European Foulbrood

**DOI:** 10.1007/s00248-022-02082-w

**Published:** 2022-08-09

**Authors:** Marylaure de La Harpe, Ayaka Gütlin, Camilo Chiang, Vincent Dietemann, Benjamin Dainat

**Affiliations:** 1 Swiss Bee Research Center, Agroscope, Bern Liebefeld, Switzerland; 2 Present address: Department of Geography, Remote Sensing Laboratories, Spatial Genetics Group, University of Zürich, Zurich, Switzerland; 3 Agroscope, Conthey, Switzerland; 4 Department of Ecology and Evolution, Biophore, UNIL-Sorge, University of Lausanne, Lausanne, Switzerland

**Keywords:** *Apis mellifera*, Bactericides, *Enterococcus faecalis*, *Melissococcus plutonius*, Resistance, Royal jelly, Social immunity

## Abstract

To defend themselves against pathogenic microorganisms, honey bees resort to social immunity mechanisms, such as the secretion of antibiotic compounds in the jelly they feed to their larvae. Whereas the bactericidal activity of jelly fed to queen larvae is well studied, little is known about the bioactivity of compositionally different jelly fed to worker larvae. However, the numerous worker larvae are likely to drive the spread of the microorganism and influence its virulence and pathogenesis. Diluted jelly or extracts are mostly used for jelly bioactivity tests, which may bias the evaluation of the pathogen’s resistance and virulence. Here, we compared the bactericidal effect of pure and diluted jellies destined for queen and worker larvae on *Melissococcus plutonius*, the etiological agent of the European foulbrood (EFB) disease of honey bees, and on a secondary invader bacteria, *Enterococcus faecalis*. We tested three strains of *M. plutonius* with varying virulence to investigate the association between resistance to antibacterial compounds and virulence. The resistance of the bacteria varied but was not strictly correlated with their virulence and was lower in pure than in diluted jelly. Resistance differed according to whether the jelly was destined for queen or worker larvae, with some strains being more resistant to queen jelly and others to worker jelly. Our results provide a biologically realistic assessment of host defenses via nutritive jelly and contribute to a better understanding of the ecology of *M. plutonius* and of secondary invaders bacteria in the honey bee colony environment, thus shedding light on the selective forces affecting their virulence and on their role in EFB pathogenesis.

**Supplementary Information:**

The online version contains supplementary material available at 10.1007/s00248-022-02082-w.

## Introduction

Because of the social organization within their colonies, honey bees can rely on innate and social immunity to defend themselves against the numerous pathogens that threaten them [1–5]. One mechanism of social immunity is derived from communal brood care expressed by honey bees. To feed larvae, adult nurse honey bees secrete nutritive jelly produced in their hypopharyngeal and mandibular glands [6]. Aside from water and sugars, this jelly contains proteins, fatty acids, and peptides with antibiotic activities [7, 8]. Because of its role in producing queens—the sole reproductive females in colonies [9]—and of the relative ease with which it can be collected or bought commercially, it is mostly the bioactivity of royal jelly that has been investigated to date [8, 10, 11]. As a result, there is a paucity of data on the bactericidal activity of jelly fed to workers [7, 12]. However, protecting the worker brood is also essential to colony survival. In addition, worker brood represents the most abundant substrate for bacterial infection and spread, hence largely determining pathogenesis and possibly influencing the pathogenicity of antagonistic microorganisms. A higher bactericidal effect of queen than worker jelly can be expected due to higher concentrations in bioactive compounds such as sugars, proteins, and fatty acids, as early as the first days of brood development [13–15].

As a result of the occurrence of bactericidal compounds, honey bee brood pathogens that transit through or multiply in the intestine of larvae must survive the jelly’s growth antagonistic or biocidal effect until they reach their replication milieu. Vegetative cells of *Paenibacillus larvae*, the pathogenic agent of the American foulbrood disease, lose their viability after a few minutes in jelly, and this pathogen relies on spores to survive the bactericidal effect of jelly before they can replicate in the larval midgut lumen and later in their hemocoel [16, 17]. *Melissococcus plutonius*, a non-spore forming bacterium causing European foulbrood disease [18], possesses vegetative cells endowed with *spxA1a* regulator gene–mediated stress resistance mechanisms to withstand the bactericidal effects of jelly until they reach the midgut lumen, where they multiply [18, 19]. The bactericidal effects of jelly have been investigated using diluted queen-destined jelly or extracts of this jelly [7], most probably to alleviate the methodological constraints generated by the high viscosity of pure jelly. However, diluted samples or extracts do not allow quantification of the antibiotic effect of jelly as it occurs in the natural situation (i.e., undiluted).

Neither the effect of worker jelly nor pure queen jelly on the survival of *M. plutonius* has been investigated to date. Differences in susceptibility to diluted queen jelly were reported between Japanese strains of *M.* *plutonius*; however, investigating a broader range of strains was deemed necessary to confirm the generality of variations in resistance to host defenses and to better establish the link between a strain’s resistance and its virulence [12]. In addition, *M. plutonius* was described as either less or more susceptible to the bactericidal effect of diluted queen jelly or to its water-soluble components than the secondary invader bacteria associated with European foulbrood, which presence is not required to trigger EFB symptoms, but which is consistently found in *M. plutonius*–infected colonies [12, 20, 21]. The ubiquitous bacterium *Enterococcus faecalis* is such a secondary invader. Its survival in larval jelly, as well as virulence, was studied in conjunction with that of *M. plutonius* [12, 20, 22] to clarify the initial uncertainties of its involvement in EFB pathogenesis [18, 23]. These uncertainties prevent a better understanding of the ecology of *M. plutonius* in the honey bee colony environment, of the selective forces affecting its virulence, and of the interactions between *M. plutonius* and secondary invaders in EFB pathogenesis [18, 21, 24, 25].

Our first aim was to determine whether three *M. plutonius* strains differing in their virulence for honey bee brood [21] and one strain of *E. faecalis* vary in their resistance to the bactericidal effect of pure worker and queen jellies. Our second aim was to evaluate the bias in bacteria survival generated by the use of 50% diluted jelly, as recommended in the standard in vitro larval assay used to study this pathogen’s pathogenicity at the individual level [e.g., 21, 22, 26, 27]. For this, we measured bacteria survival in the two jelly types and two dilutions by sampling aliquots of contaminated jelly at several time intervals and plating them on a solid culture medium. We expected that jelly fed to queens would have a higher bactericidal effect than that fed to workers and that pure jellies would have a higher bactericidal effect than diluted jellies. Because the more bacteria reach the midgut, the higher their negative impact, we also expected that *M. plutonius* virulence toward honey bee larvae would be positively correlated to its resistance to the bactericidal effect of the jellies. Finally, because *M. plutonius* is an obligate pathogen of the honey bee, we expected its resistance to the bactericidal effect of jelly to be higher than that of the ubiquitous secondary agent, *E*. *faecalis*, which is not bound to the honey bee for reproduction [28].

The measures of bacteria survival in pure worker and queen jellies and the differences in survival observed when bacteria are exposed to these two jelly types provide a biologically relevant measure of their bactericidal effect. Our results contribute to a better understanding of the role of nutritive jelly fed to immature workers and queens as a component of the social immune system of *A. mellifera* and of the evolutionary forces at play at the early stages of *M. plutonius* infection.

## Material and Methods

### Jelly Sampling

Six colonies were used for royal jelly production. The royal jelly was sampled in spring 2019 from cells containing 3-day-old queen larvae (i.e., 6 days after oviposition by the queen). Jellies from the six colonies used were mixed in a pool for the tests. The worker jelly was sampled in summer 2019 from cells containing larvae 2- to 3-day-old worker larvae from one colony. The earlier harvesting of worker jelly was due to the frequently observed larger amount of jelly deposited in the cells containing the 2-day-old larvae compared to the 3-day-old larvae. Brouwers and Beetsma [14] and Wang et al. [13] showed a stable moisture and composition in total protein, 10-HDA, fructose, glucose, free amino acids, and lipids, as well as trace elements and minerals of worker jelly until day 3 of its deposition. The effect of worker jelly collected on days 2 and 3 is thus similar and can be compared to that of the queen jelly collected solely on day 3. To facilitate worker jelly sampling, we maximized the amount of 2–3-day-old larvae by caging queens [29] on different empty combs for 24 h, 2 days in a row ahead of sampling. After removal of the larva with tweezers, worker jelly was collected by sliding the flexible tongue of a retractable Chinese grafting tool under the jelly mass and heaving it out of the cell. Antibiotic use in honey bee colonies is prohibited in Switzerland [30], and accordingly, no antibiotics had ever been used in the colonies from which the jellies were sampled and no residues could have accumulated in the hive material used.

### Bacterial Strain Cultivation

Bacterial cultivation was performed as described in [21]. Briefly, the three *M. plutonius* strains (Bailey and Collins ATCC 35311, CH 49.3, and CH Mepl*S1* (see [21])) of the Sequencing Type 3 and *E. faecalis* ((Andrewes and Horder) Schleifer and Kilpper-Balz ATCC 19433) were aliquoted out of the original stocks stored at − 80 °C in 15% glycerol to avoid recultivation and the possible associated loss of genetic material [21]. The *M. plutonius* strains were cultivated in basal medium*.* The medium contained 1% yeast extract, 1% glucose, 1% saccharose, 0.04% L-cysteine, and 0.1 M KH_2_PO_4_ in distilled water. Its pH was adjusted to 6.7 with 5 M KOH. The medium was solidified by adding 18 g.l^−1^ agar and autoclaved at 115 °C for 15 min [31, 32]. After incubation for 4 days at 36 °C under anaerobic conditions (GENbox anaer, bioMérieux), individual bacterial colonies identified as *M. plutonius* based on colony morphology were picked from the Petri dishes and inoculated in a liquid basal medium. The cultures were incubated anaerobically for another 4 days at 36 °C. *E. faecalis* was cultivated in a medium containing 10 g.l^−1^ glucose, 7.5 g.l^−1^ Bacto peptone, 6.8 g.l^−1^ KH_2_PO_4_, 2.5 g.l^−1^ yeast extract, 2 g.l^−1^ Bacto tryptone, and 2 g.l^−1^ starch. Its pH was adjusted to 7.2 with KOH.

To determine the bacterial concentration of the stock solutions of each bacterial strain or species used to spike the jelly in each experiment, these were diluted in tenfold steps. Each dilution was plated trice on solid basal medium, and the values averaged. Cultivation was performed as described above.

### Jelly Preparation and Spiking

Worker and royal jellies were used in their pure form and diluted with a sugar solution to constitute the diet used in standard larval rearing assays [27]. This sugar solution consisted of 1.2 g glucose, 1.2 g fructose, and 0.2 g yeast extract in 8.4 g filter sterilized (0.2 µm) pure water [33, 34]. This solution was then mixed in 10 g of queen jelly, resulting in a 50% (w:w) dilution of the jelly. We also mixed 10 g of worker jelly with 10 g of sugar solution to dilute it to 50% (w:w). We thereafter designated the undiluted jelly as pure, as opposed to the diluted jelly. Samples (100 μl) of each jelly type and dilution (pure and diluted queen jelly and pure and diluted worker jelly) were placed in individual Eppendorf tubes and spiked by adding 1 μl of the stock solutions of the four selected bacterial strains, followed by thorough mixing. The tubes were then immediately incubated aerobically at 34.5 °C and 98% humidity to replicate the natural conditions in larval cells and the incubating conditions of artificial rearing protocols. Subsequent aliquots of the contaminated jelly in the time series were treated in the same manner.

### Measurement of Bacteria Survival

To measure the survival rate of each bacterial strain or species in each jelly type and dilution, 10 µl aliquots of each contaminated jelly were sampled at 0, 0.5, 1, 1.5, 2, 3, and 4 h post spiking to allow for survival modeling. At each time point, the aliquot was diluted into 90 µl of NaCl 0.9% solution. The 100 µl was then halved, and each 50 μl was plated on basal medium for culture (see section “Bacterial Strain Cultivation”). These two 50 μl samples constituted technical replicates. The plates were placed in anaerobic conditions at 36 °C for 4 days to allow colony formation and subsequent counting of colony forming units (CFUs).

This procedure was performed 5 times, resulting in 1120 data points (4 strains × 2 jelly types × 2 jelly dilutions × 7 time points × 5 biological replicates × 2 technical replicates for each sample). Two of these series of experiments were prolonged with sampling at 6, 10, 18, 26, and 46 h post spiking to determine the maximum survival time of the bacteria in the jellies. Because of the smaller sample, these data were not included in the statistical analysis.

### Statistics

Because precise control of the number of bacteria used for spiking is not possible and to ensure consistent comparisons, the data were normalized by dividing the CFU per ml obtained at each time point by the initial bacterial concentration, measured immediately after jelly contamination with the bacteria. Data points with a fold change superior to 2.5 compared to the initial concentration were considered outliers and eliminated from the dataset to optimize modeling. This cutoff value was chosen as a compromise between reducing the number of outliers and retaining a maximum number of data points and resulted in the exclusion of 40 data points out of 1120 (3.6%).

To determine which factor was associated with bacterial survival, we fitted mixed linear models (lme4 package, 1.1–26 in R studio version 4.1.2 [35]) with the interaction between the fixed factors jelly type (queen vs. worker), dilution (pure vs. diluted), and bacteria strain. Given that the bacterial stock solution concentration could affect bacteria survival (antibacterial compounds in jelly could become limiting factors when stock solutions are highly concentrated), this variable was nested within the corresponding experiment and considered a random factor. Given the magnitude difference compared to the survival counts, the bacteria stock solution concentration used to contaminate the jellies was log transformed before analysis. Finally, because the samples were obtained from the same pool of contaminated jelly sampled over time, the principle of independence within a replicate was violated, and we thus considered the sampling time a random factor. Model selection was achieved by removing each factor from the initial full model. The final model was selected based on its relative maximal likeliness (REML), for which the lowest value indicates the best fit to the data. The significance of the selected factors was analyzed using a three-way ANOVA (mixlm package, 1.2.4 in R studio version 4.1.2). The graphs were produced using R Studio version 4.1.2.

## Results

**Fig. 1:**
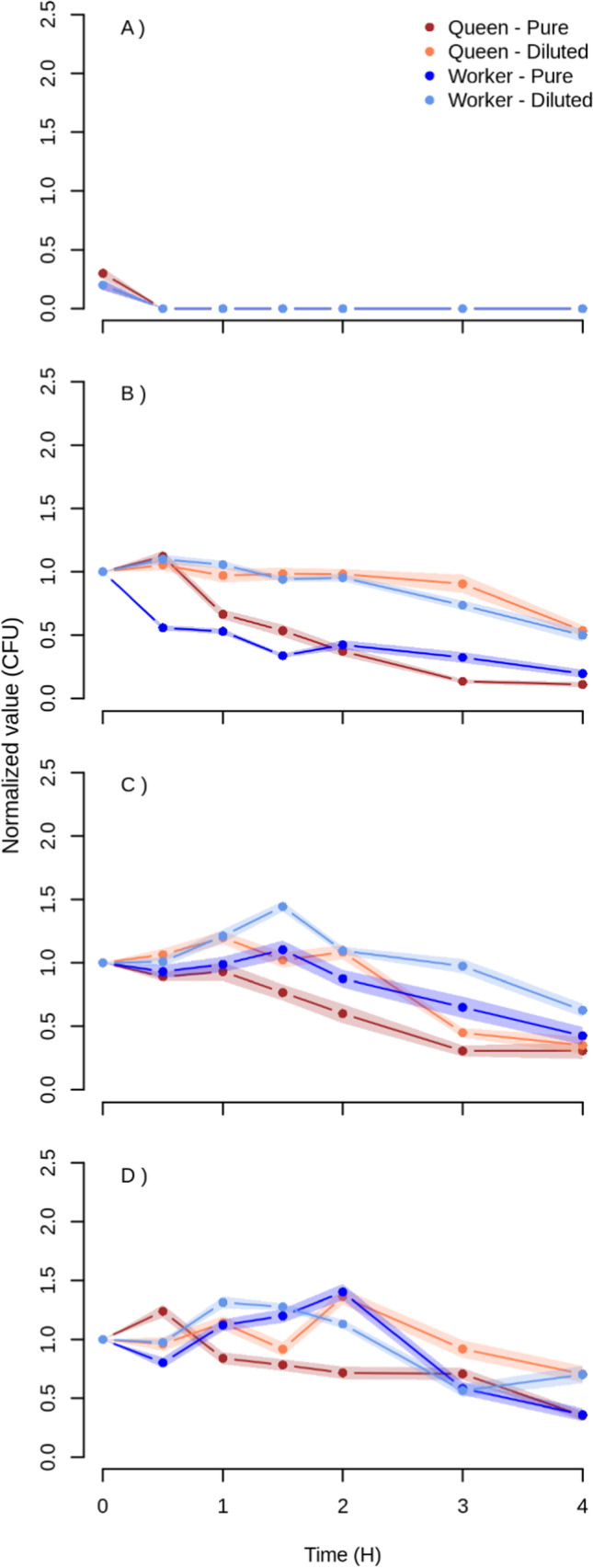
Survival of the tested bacterial species and stains expressed as fold change of the initial concentration for *Enterococcus faecalis* (**A**), *Melissococcus plutonius* ATCC (**B**), *M. plutonius* CH49.3 (**C**), and *M. plutonius* CH MeplS1 (**D**). Shaded areas correspond to standard errors

We observed a decline in survival of all strains/species tested within 26–46 h (Fig. S1). *E. faecalis* perished rapidly once placed in both pure and 50% diluted jelly of both types (Fig. 1A). As no colony was observed after 30 min, this species was not included in the modeling of bacterial survival.

The *M. plutonius* reference strain *(*ATCC 35,311*,* Fig. 1B) showed a gradual decline in survival over time in all jelly types and dilutions. The decline was, however, faster in pure jelly, independent of the type of jelly (worker or queen). The fold change reduction in CFUs within 4 h amounted to 9.1 and 1.9 for queen pure and diluted jelly and to 5.3 and 2.0 for worker pure and diluted jelly, respectively. The survival pattern of *M. plutonius* strains CH 49.3 and CH MeplS1 was irregular, with CFUs even increasing under most experimental conditions (Fig. S1 C and D). The best-fitted model included the fixed factors bacterial strain and jelly dilution, with the interactions of bacterial species with jelly dilution and type, and the random factors time and stock solution concentration (Table 1).

**Table 1:** Output of the three-way ANOVA model with the best fit using the replicate as a random effect nested in time and the initial bacterial concentration nested in each experiment. Significant *p*-values are indicated in bold

	Chisq	Df	Pr (> Chisq)
Intercept	223.8618	1	** < 2.2 × 10**^**–16**^
JellyDilution	8.5931	1	**0.003374**
JellyType	0.0075	1	0.93
BacterialStrain	19.3928	2	**6.1 × 10**^**–05**^
JellyDilution: BacterialStrain	11.6375	2	**0.002971**
JellyType: BacterialStrain	7.8921	2	**0.019331**

The fixed factors jelly dilution and bacterial strains had significant effects on bacterial survival, and the strains interacted significantly with both jelly dilution and jelly type (Table 1). Because of the latter, the jelly type was retained in the model despite its lack of significant effect on bacteria survival (Table 1).

## Discussion

Pure queen and worker jellies had a higher bactericidal effect on *M. plutonius* compared to their 50% dilution. The various *M. plutonius s*trains tested differed significantly in their ability to resist the bactericidal effect of the jellies, and their degree of resistance depended on jelly dilution and type (i.e., whether queen- or worker-destined), as shown by the interaction terms in the survival model. Survival of all *M. plutonius* tested for exposure to jellies was much higher than that of the secondary agent commonly found in EFB-diseased colonies, *E. faecalis*.

In line with previous work [12], the secondary invader bacteria *E. faecalis* succumbed within 30 min of exposure to the jellies despite its general high resistance to adverse conditions [36]. This rapid loss of viability can explain why *E. faecalis* does not contribute to European foulbrood pathogenicity [22] and suggests that, to be commonly found in symptomatic larvae, this bacterium is continuously brought into brood cells by contaminated honey bee nurses. By contrast, the *M. plutonius* bacteria of all strains remained viable for several hours. CH 49.3 and CH MeplS1 even multiplied in both pure and diluted jellies (Fig. 1, Fig. S1), as already observed in diluted royal jelly [12]. The higher resistance of *M. plutonius* to the bactericidal effect of diluted queen jelly compared to *E. faecalis* [12] was observed here to also occur in pure queen and worker jellies. Given that *M. plutonius* is an obligate pathogen of *A. mellifera* and is a non-spore-forming bacterium, this high resistance is essential to its survival. This high longevity ensures that a proportion of the bacteria contaminating the jellies remain viable until they are ingested by the larvae and reach their host’s midgut, where they replicate. In most instances, viable bacteria were not detected after 26 h, but in four cases out of 24, colonies were observed after a 46-h exposure to jelly (Fig. S1), indicating that they have a probability of successfully infecting their host even after staying over a day in the hostile jelly environment.

*E. faecalis* is ubiquitous and can be found in many other matrices outside a honey bee colony [28]. The selective pressure to adapt to the bactericidal effect of jellies is thus absent or reduced, leading to the rapid disappearance of *E. faecalis* from the jelly [24, 25]. However, Vezeteu et al. [20] found that *M. plutonius* experienced a higher negative effect from exposure to water extracts of royal jelly than *E. faecalis*. This difference in findings is likely due to different experimental methods used, especially to the type of solutions in which the jellies were diluted (water vs. broth and sugar solution) and to the different end-points measured (bacterial growth vs. survival). It is thus possible that water extracts inhibit *M. plutonius* growth to a higher degree compared to *E. faecalis*, whereas jelly diluted in broth or sugar solution kills more *E. faecalis* than *M. plutonius* bacteria. This distinction highlights the need to test the bactericidal effect of jellies under more biologically relevant and standardized conditions.

As expected from the dose-dependent effect of diluted royal jelly or of particular bactericidal components shown in previous studies [12, 20], the bactericidal effect of pure jelly was significantly higher than that of 50% diluted jelly. This finding calls for caution in interpreting the effect of brood pathogens when they are measured in assays using diluted queen jelly as a larval diet (e.g., [33]). The higher survival of the bacteria in the diluted jelly used as larval diet could lead to increased negative effects on the host compared to the natural situation and to an overestimation of its virulence. Furthermore, our model of bacterial survival showed a significant interaction between bacterial strain and jelly dilution, i.e., with the concentration of antibacterial compounds, indicating that the survival of the strains varied according to jelly dilution and pointing to differences in the resistance mechanisms between strains [19].

The factor strain had a significant effect in the model of *M. plutonius* survival, indicating that the three strains tested differed in their resistance to the jellies. The number of viable bacteria belonging to the reference strain ATCC 35,311 gradually decreased, while that of CH 49.3 and CH MeplS1 appeared more resistant, with even some growth observed at some time points. These results complement those of a previous study performed on Japanese strains of *M. plutonius* [12] and support the general occurrence of such differences outside of Japan, at least for strains of the sequencing type 3 of clonal complex 3, to which CH 49.3 and CH MeplS1 belong [21]. According to previous work, strain CH 49.3 has a high virulence, CH MeplS1 is avirulent, and the reference strain ATCC 35,311 has medium virulence [21, and unpublished data]. For CH 49.3 and ATCC 35,311, there was an inverse correlation between survival ability in jelly and the degree of resistance to bactericidal compounds, which supports the idea that resistance to the jelly’s bactericidal compounds contributes to their degree of virulence [12]. This trend did not extend to the avirulent CH MeplS1, which is likely due to the close genetic proximity to CH 49.3. MeplS1 is a culture derivative of CH 49.3, which lost genes related to virulence during the cultivation step [21, 37]. These lost genes thus did not affect resistance to bactericidal compounds in the jellies, in line with previous work [19]. More strains differing in virulence and sequencing types should be compared to confirm the relationship between virulence and resistance to jelly bactericides. The mechanisms underlying bacterial resistance to jelly and their virulence should be identified to obtain a better understanding of the potential arms race between the host and pathogen.

The bactericidal activities of queen and worker jellies were significantly different depending on the bacterial strain used (Table 1). The significance of this interaction combined with the non-significance of the factor jelly type alone, may indicate a crossover or qualitative interaction [38]. Some bacterial strains are thus more susceptible to queen- than to worker-destined jelly, as previously shown for a strain of *P. larvae* [39], whereas others show the opposite pattern. This difference in susceptibility suggests that some strains are better adapted to infect queen larvae and other to infect worker larvae. These results indicate a more complex pathogen–host relationship than recognized to date and warrant further studies. The standard in vitro larval rearing assays using royal jelly [33] may fail to detect such effects and thus only provide a partial picture of the host–pathogen interaction. A variant of this method using worker jelly and pure jellies is desirable to better capture its complexity. A further factor to consider is the differences in jelly composition between colonies that may lead to different abilities to resist infections by this and other pathogens, which need to transit in jelly to reach their replication milieu.

## Conclusion

*M. plutonius* resists for several hours the bactericidal environment of queen and worker jellies, which are its obligated route of infection of their only host, the young honey bee larvae. This ability may determine their virulence, which appears to vary according to the caste of the host they infect. Further tests of these hypotheses should be performed in conditions reflecting the pathogen’s natural environment, which requires the development of adapted tools. Only in such biologically relevant conditions will we be able to replicate the selective forces acting on this pathogen and its host and accurately identify respective adaptations.

## Supplementary Information

Below is the link to the electronic supplementary material.

Supplementary file1 (DOCX 147 KB)

## Data Availability

The dataset generated during the current study is available from the corresponding author on reasonable request.
