# Supplementary material for: Influence of Honey bee Nutritive Jelly Type and Dilution on its Bactericidal Effect on Melissococcus plutonius, the Etiological Agent of European Foulbrood
Source: Microb Ecol. 2022 Aug 9;86(1):617–23. doi: 10.1007/s00248-022-02082-w (PMC10293366; doi:10.1007/s00248-022-02082-w)
Supplement: Supplementary file 1 — Supplementary file1 (DOCX 147 KB) [file 248_2022_2082_MOESM1_ESM.docx]

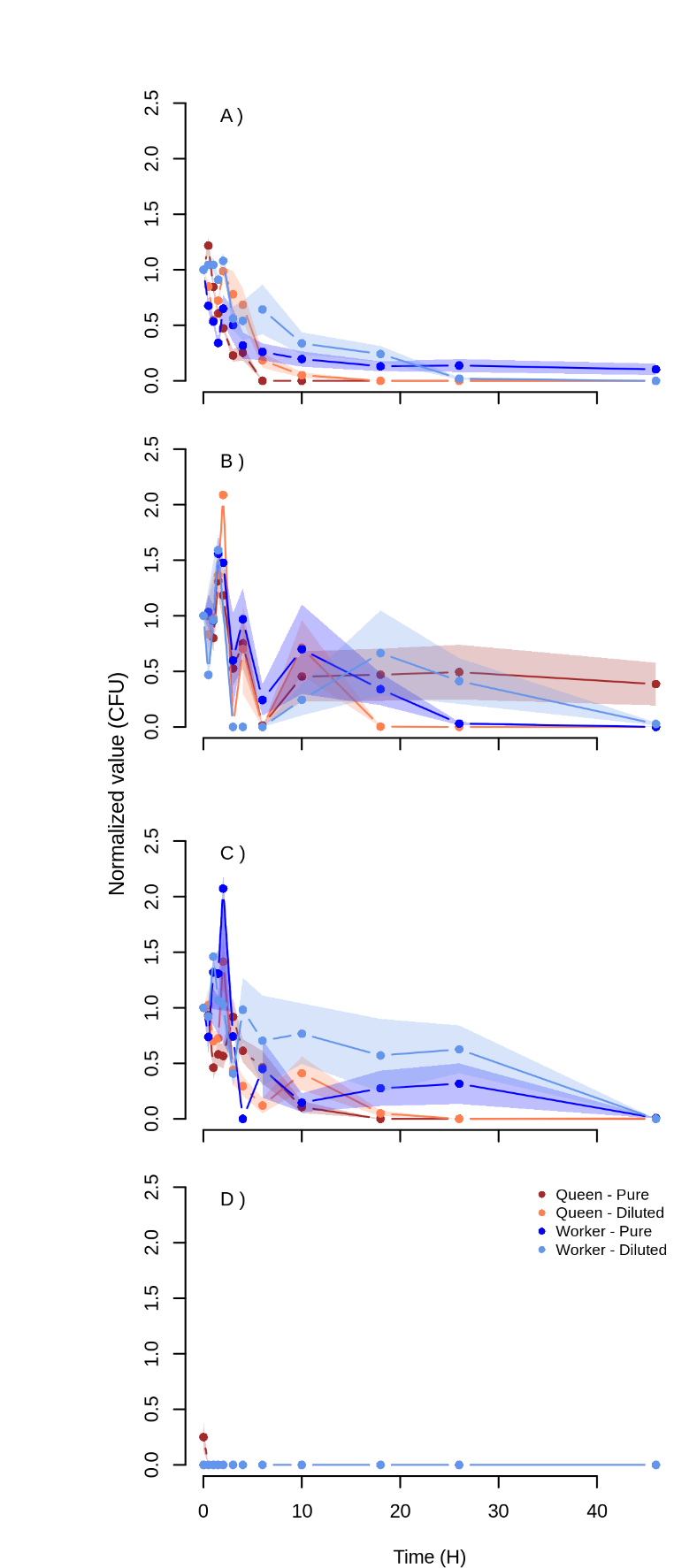


**Supplementary Fig. 1** Average survival of the tested bacterial species and stains expressed as fold change of the initial concentration over time for *Melissococcus plutonius* ATCC (A), *M. plutonius* CH49.3 (B), *M. plutonius* CH MeplS1 (C), and *Enterococcus faecalis (D),* with values up to 46 hours after inoculation in two different experiments. Shaded areas correspond to standard errors.
